# Targeting tissue factor as a novel therapeutic oncotarget for eradication of cancer stem cells isolated from tumor cell lines, tumor xenografts and patients of breast, lung and ovarian cancer

**DOI:** 10.18632/oncotarget.13644

**Published:** 2016-11-26

**Authors:** Zhiwei Hu, Jie Xu, Jijun Cheng, Elizabeth McMichael, Lianbo Yu, William E. Carson

**Affiliations:** ^1^ Department of Surgery, Division of Surgical Oncology, The Ohio State University Medical Center and The James Comprehensive Cancer Center, Columbus, OH, USA; ^2^ Yale University School of Medicine Department of Obstetrics, Gynecology and Reproductive Sciences, New Haven, CT, USA; ^3^ Biomedical Sciences Graduate Program, The Ohio State University Medical Center and The James Comprehensive Cancer Center, Columbus, OH, USA; ^4^ Center for Biostatistics, Department of Biomedical Informatics, The Ohio State University Medical Center and The James Comprehensive Cancer Center, Columbus, OH, USA

**Keywords:** cancer stem cells, solid cancer, tissue factor, targeted immunotherapy, targeted photodynamic therapy

## Abstract

Targeting cancer stem cell (CSC) represents a promising therapeutic approach as it can potentially fight cancer at its root. The challenge is to identify a surface therapeutic oncotarget on CSC. Tissue factor (TF) is known as a common yet specific surface target for cancer cells and tumor neovasculature in several solid cancers. However, it is unknown if TF is expressed by CSCs. Here we demonstrate that TF is constitutively expressed on CD133 positive (CD133+) or CD24-CD44+ CSCs isolated from human cancer cell lines, tumor xenografts from mice and breast tumor tissues from patients. TF-targeted agents, i.e., a factor VII (fVII)-conjugated photosensitizer (fVII-PS for targeted photodynamic therapy) and fVII-IgG1Fc (Immunoconjugate or ICON for immunotherapy), can eradicate CSC via the induction of apoptosis and necrosis and via antibody-dependent cellular cytotoxicity and complement-dependent cytotoxicity, respectively. In conclusion, these results demonstrate that TF is a novel surface therapeutic oncotarget for CSC, in addition to cancer cell TF and tumor angiogenic vascular endothelial TF. Moreover, this research highlights that TF-targeting therapeutics can effectively eradicate CSCs, without drug resistance, isolated from breast, lung and ovarian cancer with potential to translate into other most commonly diagnosed solid cancer, in which TF is also highly expressed.

## INTRODUCTION

Cancer stem cells (CSC) are a small subpopulation of neoplastic cells within a tumor that theoretically possess the capacity to self-renew and develop into the heterogeneous lineages of cancer cells that comprise the tumor [1]. The CSC hypothesis not only offers a model of tumorigenesis, but also helps explain tumor heterogeneity, recurrence and resistance to therapeutic agents [1–3]. Recent reports suggest that CSC may contribute to tumor angiogenesis, resistance to multiple therapies [4, 5] and metastasis [3, 4, 6]. There is a need to develop therapies that can specifically target and eradicate CSC, so that cancer can be treated at its earliest stages. The challenge is to identify selective surface therapeutic targets for CSCs.

Tissue factor (TF) is a 47-kDa membrane-bound cell surface receptor [7–9] and can initiate blood coagulation upon disruption of vessel wall integrity [10, 11]. TF is a common yet specific biomarker for cancer cells and tumor vascular endothelial cells in solid cancers [12–15]. Using vascular endothelial growth factor (VEGF)-induced *in vitro* angiogenic vascular endothelial models, we showed that TF is an angiogenic-specific receptor and the target for factor VII-targeted therapeutics [16]. It is unknown if TF is consistently expressed by CSC. We hypothesize that TF can serve as a novel biomarker for CSC and propose that targeting TF represents a novel therapeutic approach for the eradication of CSC.

To target TF-expressing angiogenic vascular endothelial cells (VEC) and cancer cells, Dr. Garen and Dr. Hu co-invented and developed two therapeutics using fVII, the natural ligand for TF, as the targeting domain in the context of immunotherapy [13, 14, 17] and photodynamic therapy (PDT) [15, 18–20]. For TF-targeted immunotherapy, Hu et al. constructed an immuno-conjugate of active site-mutated fVII and human IgG1 Fc (fVII-IgG1Fc), called ICON [13, 14, 17]. Intra-lesional ICON immunotherapy of experimental melanoma, prostate and head and neck tumors leads to marked tumor inhibition, and in some cases, complete eradication without affecting normal tissues [13, 14, 17, 21]. Upon binding to TF-expressing cancer cells, ICON can mediate natural killer cell (NK) cell dependent antibody-dependent cell-mediated cytototoxicity (ADCC) and complement-dependent cytotoxicity (CDC) as its mechanism of action [21]. For TF-targeted PDT, Hu et al. conjugated a monomeric fVII peptide with the photosensitizers (PS) verteporfin (VP) and Sn(IV) chlorin e6 (SnCe6) (referred to as fVII-VP and fVII-SnCe6, respectively) and showed that fVII-targeted PDT could selectively and effectively kill angiogenic vascular endothelial cells and cancer cells *in vitro* and *in vivo* in mouse models of human breast [18–20] and lung cancer [15].

To test our central hypothesis in the clinical realm, we assessed the impact of the CSC-killer drugs on putative stem cells isolated from cancer cell lines, tumor xenografts from mice as well as from human tumors of various types, including triple negative breast cancer (TNBC), lung cancer and ovarian cancer. TF is highly expressed in these cancer cells (80%-100% in breast cancer, 40%-80% in lung cancer and 84% in ovarian cancer) [15]. These three types of cancer are not only difficult to control, but also are major causes of mortality in the United States and worldwide and often develop CSC-based resistance to chemotherapy and radiation therapy [22–24]. Our marker for isolation of CSC was CD133 (AC133), which has been confirmed as a cancer stem cell marker [1, 2] in cancer of the brain, colon, breast, lung, ovaries, head and neck and pancreas. The CSC marker CD133 has been reported to co-express with another CSC marker, CD44, in ovarian cancer and hepatocellular carcinoma [25, 26]. So their expression of TF and CD44 were also examined. Their tumor initiating ability was verified by a tumorsphere assay *in vitro* and by tumor xenograft assay *in vivo* in severe combined immunodeficiency (SCID) mice [1]. Finally the efficacy and mechanism of action of ICON and fVII-tPDT were tested *in vitro* for the eradication of CSCs with comparisons to non-CSC cancer cells.

## RESULTS

### TF is expressed by CD133+ CSCs isolated from human cancer lines, tumor xenografts and patients' tumor tissues

To obtain putative stem cells for identification of novel CSC biomarkers, CD133+ cancer cells were isolated from various human tumor cell lines, including MDA-MB-231 Triple-negative breast cancer (TNBC), H460 and A549 (lung cancer), OVCAR-5 and HEY (ovarian cancer), from subcutaneous human lung tumor xenografts established in immunodeficient mice and from surgically resected primary breast tumor tissues from six patients. The results in Supplementary Table S1 verified that CD133+ CSCs represent a small population in cultured cancer cell lines (0.1% to 2%), tumor xenografts (0.5% for H460 and A549, 3% for MDA-MB-231) and tumor tissues from patients with breast cancer (3.82%). These isolated CD133+ cancer cells were further verified that they were tumor initiating stem cells, i.e., CSCs, by *in vitro* tumorsphere assays in 96 well micro-plates and by *in vivo* tumor xenograft assays in SCID/Beige mice.

The results of *in vitro* tumorsphere assays demonstrated that CD133+ cancer cells formed significantly more (*p* values <0.05 or less for CD133+ vs. CD133-) (Supplementary Figure S1 and Supplementary Table S2) and larger (Supplementary Figure S2) tumorspheres than CD133- cancer cells did. We also tested whether the isolation protocol and the medium in the sphere assays would cause variations of sphere size and/or number. The results (Supplementary Figure S1-S2 and Supplementary Table S2) showed that there were no significant differences in tumorsphere numbers between two CD133 antibody-based isolation protocols (EasySep vs. MACS) and between two tumorsphere assay media (FBS-containing growth medium vs. serum-free stem cell medium) (Supplementary Table S2 and Supplementary Figure S1).

The *in vivo* tumor xenograft assays showed that even 100 or 200 CD133+ H460 cancer cells could form significantly larger tumors that were detectable earlier than those CD133- H460 cells (results with 200 cells reported in Supplementary Figure S3; results with 100, 500 and 1000 cell groups reported in Supplementary Figure S4). Supplementary Figure S3a showed that 200 CD133+ H460 cells formed tumors with an average size of 60 mm^3^ on day 17, whereas CD133- cells took almost twice as long (31 days) to form the same size tumor (60 mm^3^) (p<0.0001 from day 17 through day 38). The sizes and weights of tumors formed by CD133+ cancer cells were also greater than those from CD133- cancer cells in the same mouse groups, as observed morphologically (Supplementary Figure S3b and S3c) and quantitatively (CD133+ cells-derived tumor weighed at 0.73 ± 0.39 grams vs. CD133- cell-derived tumor weighed at 0.10 ± 0.15 grams, *p*=0.0302) (Supplementary Figure S3d).

**Figure 1:**
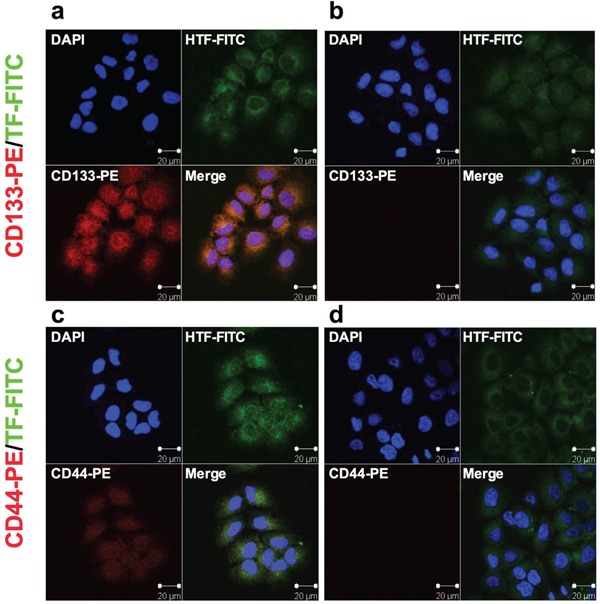
TF expression in human lung cancer H460 CD133+ CSCs isolated from *in vitro* cultured cancer cell line **a-d.** CD133+ CSCs (a, c) and CD133- non-CSCs (b, d) were immunofluorescently stained for expression of CD133 (red) and TF (green). Their nuclei were stained by DAPI (blue) and the cells were photographed under confocal microscopy (Zeiss). Scale bar: 20 μm.

**Figure 2:**
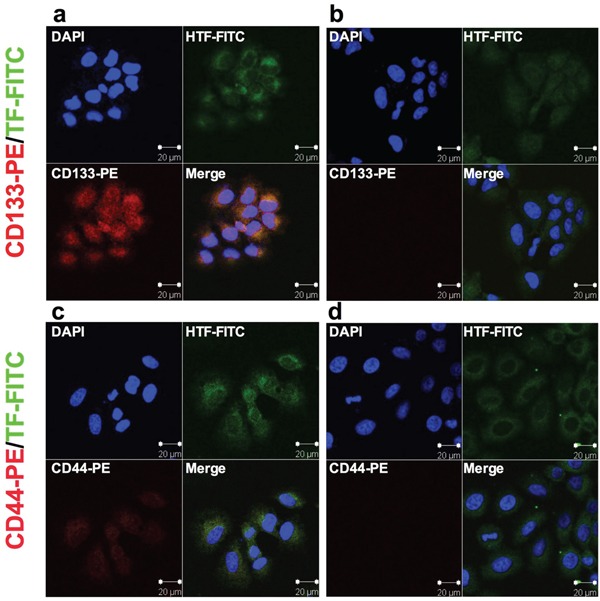
TF expression on lung cancer H460 CD133+ CSCs isolated from subcutaneous human lung tumor xenografts in mice **a-b.** Representative confocal microscopy imaging of co-expression of TF and CD133 on CD133+ CSCs (a) and only TF expression on CD133- non-CSCs (b) isolated from H460 tumor xenografts. **c-d**. Representative imaging of co-expression of TF and CD44 on CD133+ CSCs (c) and only TF expression on CD133- non-CSCs (d). Scale bar: 20 μm.

**Figure 3:**
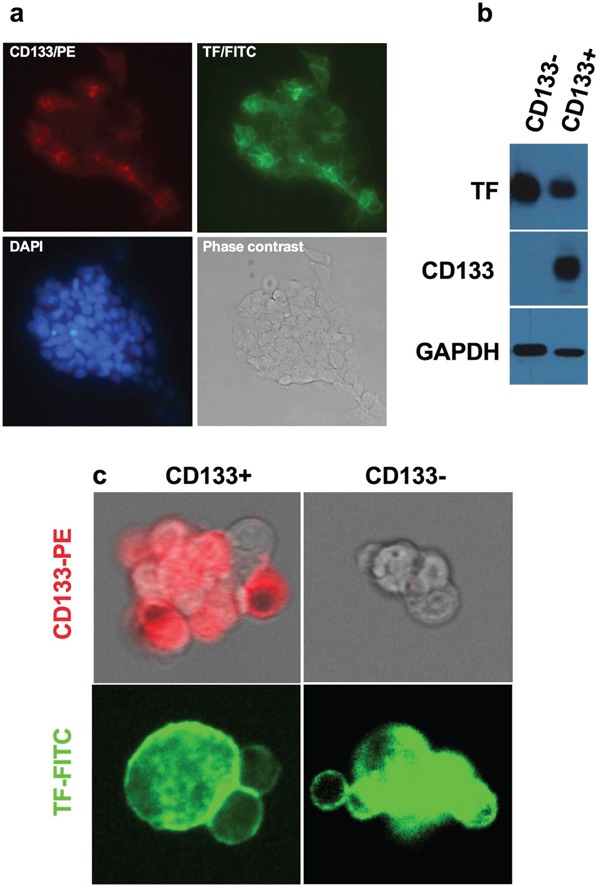
TF expression on CD133+ CSCs isolated from OVCAR-5 line, MDA-MB-231 line and from patients' breast tumor tissues **a.** Representative imaging of TF expression on CD133+ CSC OVCAR-5 cells. Original magnification: 200 × **b.** Immunoblotting for TF expression on CD133+ CSCs and CD133- non-CSC MDA-MB-231 cells. CD133 expression was confirmed on CD133+ CSCs and GAPDH was for assessing sample loading. **c.** Representative imaging of TF expression on breast cancer patients' CD133+ CSCs and CD133- non-CSCs, CD133 expression was confirmed only on CD133+ CSCs, not on CD133- cells (Original magnification: 25 μm under ZEO Fluorescent Cell Imager, Bio-Rad).

Having verified that CD133+ cancer cells are CSCs, we examined expression of TF and/or CD44 on CD133+ CSCs isolated from the human cancer lines, tumor xenografts and patients' tumor tissues to identify new biomarkers for CSCs. Figure 1 shows co-expression of TF and CD44 in CD133+ cancer cells isolated from the human lung cancer line H460, i.e., CD133+ cells from H460 line co-express TF (Figure 1a) and CD44 (Figure 1c), whereas CD133- cells express only TF (Figure 1b and 1d). Similarly, Figure 2 shows co-expression of TF and CSC markers (CD133 and CD44) in CD133+ H460 cells (Figure 2a and 2c) isolated from human tumor xenografts grown in mice, whereas CD133- cancer cells express only TF, but no CD44 and CD133 (Figure 2b and 2d). Figure 3 shows that CD133+ cancer cells, either from the ovarian cancer line OVCAR-5 (Figure 3a), TNBC MDA-MB-231 line (Figure 3b), or from breast cancer patients (Figure 3c), all express TF. CD133- breast cancer cells from *in vitro* cultured line (Figure 3b) and from patients (Figure 3c) also express TF. In summary, both the CD133+ and CD133- cancer cells express TF, either isolated from cancer cell lines, tumor xenografts or from patients' tumor tissues.

To further validate TF expression on CSCs, we also used a CD44-based human CD24 negative CD44 positive (CD24- CD44+) breast cancer stem cell isolation protocol to isolate CD24- CD44+ CSCs from the TNBC line (MDA-MB-231) and patient-derived human breast cancer cells. It was observed that the CD24- CD44+ CSCs were also positive for expression of CD133 and TF, whereas non-CSC CD24- cancer cells were negative for express of CD133 (not shown).

### TF-targeted PDT using fVII-conjugated photosensitizer SnCe6 is effective in eradicating CSCs

**Figure 4:**
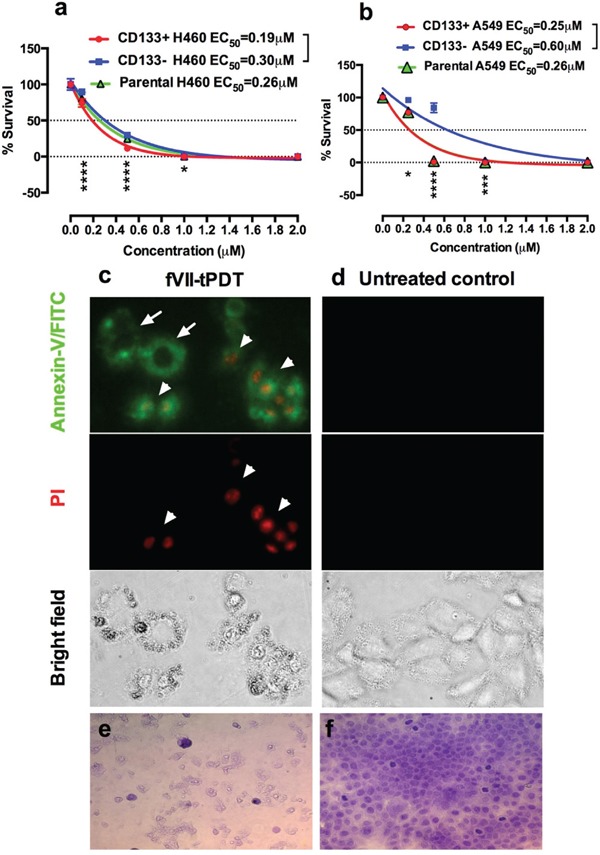
TF-targeted PDT with fVII-SnCe6 is effective in eradicating CD133+ CSCs and CD133- non-CSC cancer cells by inducing apoptosis and necrosis **a-b.** % survival of CD133+ CSCs, CD133- non-CSC and parental cancer cells H460 (a) and A549 (b) after being treated by fVII-tPDT for 36 J/cm^2^ 635nm laser light, as determined by clonogenic assay. *: p<0.05; **: p<0.01; ***: p<0.001; ****: p<0.0001 by linear mixed model with t-test. **c-d.** After fVII-tPDT, the CD133+ H460 CSC cells (c) were stained with Annexin V-FITC and then stained with propidium iodide (PI). Untreated CD133+ H460 CSCs were the control cells (d). The cells were photographed under a fluorescent microscope using green (FITC), red (PI), and phase channels. Original magnification: 200 ×. **e-f.** Representative imaging of CD133+ CSCs from OVCAR5 line were stained with crystal violet dye after fVII-tPDT (e), and the control CSCs (f) were not treated. Original magnification: 400 ×. Data presented as Mean ± SEM %. The results in a-b were repeated 2-4 times per cancer line for a total of 10 independent experiments. The mechanism studies in c-d were representative from two independent experiments with H460 line and repeated one time in OVCAR5 line. Observations on cellular destruction by fVII-tPDT in e-f were representative from two independent experiments with OVCAR5 and were reproducible in other cancer lines (Supplementary Figure S6).

Having shown that TF is expressed by CD133+ CSCs, we could use these cells as an *in vitro* model to study the eradiation of CSC-based tumors. As a step towards eradicating CSC-based tumors by TF-targeted PDT using fVII-conjugated SnCe6, we examined the effectiveness of fVII-tPDT for killing CD133+ TF+ CSCs *in vitro* in this study. Figure 4 shows that fVII-tPDT effectively eradicated CD133+ H460 CSCs (Figure 4a) and A549 CSCs (Figure 4b) isolated from lung tumor xenografts. The doses of fVII-SnCe6 conjugate and laser energy tested for CSCs were based on our published data on the parental H460 and A549 cancer lines [15]. With 1 μM SnCe6 and 36 J/cm^2^ laser energy, fVII-tPDT was able to completely eradicate CD133+ H460 and A549 CSCs (0% survival), whereas the survival of CD133- non-CSC H460 and A549 cancer cells was 0.3% (*p*<0.001 for CD133+ vs. CD133- H460, Supplementary Table S3). When increasing SnC6 to 2 μM, fVII-tPDT was able to completely eradicate CD133+ CSCs, CD133- non-CSCs and parental H460 and A549 lung cancer cells (Figure 4a and 4b and Supplementary Table S3). We have previously shown that fVII-PS alone and laser light alone did not have effects in killing cancer cells [20]. FVII-targeting significantly improved not only the effectiveness and but also the selectivity of non-targeted PDT effect in killing lung and breast cancer cells and angiogneic vascular endothelial cells *in vitro* [15, 20], all of which expressed TF. In contrast, fVII-tPDT had no effect on TF-negative normal cell controls, including 293 [20], CHO-K1 [21] and unstimulated HUVEC (a model of quiescent endothelial cell control) [20, 21]. Moreover, it was verified that the effect of fVII-tPDT was mediated by fVII binding to TF [20]. Thus we believe that eradication of CSCs by fVII-tPDT was mediated specifically via TF targeting.

To compare the effectiveness of fVII-tPDT for killing parental, CD133+ CSC and CD133- cancer cells, half maximal effective concentrations (EC_50_) of SnCe6 were determined using One-phase decay (Prism software). Figure 4a showed that, with laser energy at 36 J/cm^2^, EC_50s_ of SnCe6 in fVII-SnCe6 conjugate were 0.19 μM, 0.30 μM and 0.26 μM for CD133+ CSCs, CD133- non-CSCs and parental H460 cancer cells, respectively. Similarly, EC_50s_ of SnCe6 in fVII-tPDT for CD133+ CSCs, CD133- non-CSCs and parental A549 cancer cells were 0.25 μM, 0.60 μM and 0.26 μM, respectively (Figure 4b). The EC_50s_ for CD133- and parental A549 cells are similar to the published data for the same cancer line [15]. Statistical analyses showed that CD133+ CSCs was significantly more sensitive to fVII-tPDT than CD133- non-CSC cancer cells (*p* values <0.01 or less) (Figure 4a and 4b, Supplementary Table S3), suggesting that CSCs are actually more sensitive to fVII-tPDT treatment than non-CSC cancer cells.

TF-targeted PDT was also effective in killing human ovarian CSCs. The CSCs from OVCAR5 (Supplementary Figure S5a) and HEY cells (Supplementary Figure S5b) were more sensitive to fVII-tPDT (2 μM SnCe6 in fVII-SnCe6 and 36 J/cm^2^) than CD133- non-CSC cancer cells (*p*=0.0107 and *p*=0.0437, respectively). It is worth noting that fVII-tPDT was able to completely eradicate CSC from ovarian cancer cells. To verify that those CSC had been killed after fVII-tPDT treatment, we carried out both the clonogenic assay and the crystal violet staining assay for determining the effect of fVII-tPDT. Supplementary Table S4 showed that after fVII-tPDT treatment (2 μM SnCe6 in fVII-SnCe6 and 36 J/cm^2^), both the CD133+ CSCs and CD133- cancer cells were completely eradicated (0% survival) as determined by the clonogenic assay, whereas the membrane-staining assay, due to its limitations that crystal violet would stain any cellular membrane, either viable cells or cell debris (see Figure 4e–4f and Supplementary Figure S6), so that the membrane staining assay showed that the survival % of CD133+ CSCs was ~31% and CD133- H460 was ~40% (*p*=0.0171). Taken together, these results suggest that fVII-tPDT can completely eradicate CD133+ CSCs and CD133- non-CSC cancer cells without drug resistance.

To elucidate the mechanism of action of fVII-tPDT in killing CD133+ CSCs, we stained the CSC for evidence of apoptosis and necrosis immediately after fVII-tPDT treatment. Figure 4c shows that fVII-tPDT induced apoptosis (Annexin-V/FITC stained membrane) and necrosis (PI stained nuclei) in CD133+ H460 CSCs. Under bright field, it is obvious that the membrane integrity of H460 CSCs was damaged due to the fVII-tPDT treatment (Figure 4c), whereas untreated CSC control cells showed no signs of apoptosis or necrosis (Figure 4d). The observation about CSC was similar to those in CD133- and parental H460 cancer cells (Supplementary Figure S5c). Crystal violet staining assay further verified the cellular destruction of OVCAR5 CSCs by fVII-tPDT (Figure 4e), whereas untreated control cells were intact and confluent (Figure 4f). The observations on CSC cellular destruction by fVII-tPDT were repeatedly observed in CSCs from lung cancer A549 (Supplementary Figure S6) and H460 (not shown) and were consistent with our observations in the H460 cancer cells [15].

### ICON effectively kills CSCs via antibody-dependent cell-mediated cytotoxicity (ADCC) and complement-dependent cytotoxicity (CDC)

**Figure 5:**
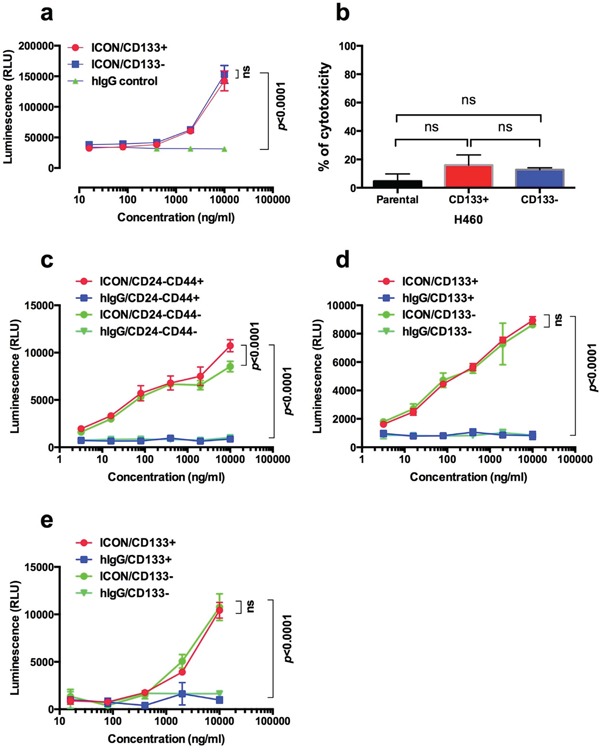
TF-targeted ICON is effective in mediating ADCC and CDC to kill CSCs and non-CSC cancer cells **a.** ICON-dependent NK-mediated ADCC was effective in killing CD133+ CSCs and CD133- non-CSC H460 cells; **b.** ICON-mediated CDC to CD133+ CSCs and non-CSC H460 cells. **c-d.** ICON can kill CD24-CD44+ CSCs (c) and CD133+ CSCs (d), as well as non-CSCs (CD24-CD44- in c and CD133- in d), isolated from MDA-MB-231 line separately using CD24- and CD44-based breast cancer stem cell kit and CD133-PE positive selection protocol. **e.** ICON-mediated ADCC to CSCs (CD133+) and non-CSCs (CD133-) breast cancer cells isolated from breast cancer patients. *p* values were analyzed by linear mixed model (a, c, d, e) or ANOVA model (b) with t-test. Data presented as Mean ± SEM. Each experiment was repeated 2-3 times.

In addition to testing TF-targeted fVII-tPDT for eradicating CSCs, we tested another TF-targeted agent, namely ICON (fVII-IgG1Fc), for killing CSC via CD16+ effector cell- and complement-mediated immunotherapy. Figure 5 shows that ICON was effective in killing CSCs via ADCC and CDC. ICON could augment CD16+ effector cell-mediated ADCC to kill CD133+ CSCs and CD133- non-CSC H460 lung cancer cells (no significant difference between CD133+ and CD133- cells) (Figure 5a), whereas human IgG control antibody showed no ADCC effect on those cancer cells (*p*<0.001 between ICON and IgG control). ICON could also activate complement to kill CD133+ CSCs, CD133- and parental H460 cancer cells (Figure 5b).

To verify that ICON-mediated ADCC is also effective in killing CD24-CD44+ CSCs isolated from the CD24 and CD44-based CSC isolation protocol, we compared the effect of ICON-mediated ADCC assay in killing CD24-CD44+ and CD133+ CSCs isolated from the TNBC MDA-MB-231 line and breast cancer CSCs isolated from patients. Figure 5c and 5d shows evidence that ICON was equally effective in killing CSCs and non-CSC breast cancer cells, using either CD24 CD44 antibodies-based protocol (R&D) (Figure 5c) or CD133 antibody-based protocol (StemCell) (Figure 5d). Figure 5e shows that ICON was equally effective in killing CD133+ CSCs and CD133- non-CSC breast cancer cells isolated from surgically dissected primary tumor tissues from patients, while IgG isotype negative control showed no effect in killing any cancer cells (*p* value not significant for CSCs vs. non-CSCs; *p*<0.0001 between ICON and IgG isotpye control for CSCs and non-CSCs).

## DISCUSSION

Dr. Wicha's group has elegantly summarized and proposed to target CSC via CSC surface markers, such as ALDH (aldehyde dehydrogenase), CD44, CD133 and HER2, or the CSC niche, such as tumor associated macrophages and myeloid-derived suppressor cells, using immunologic approaches [27]. In this study, we identified TF, a membrane-bound receptor that is different from those previously known CSC targets, as a novel surface therapeutic target for CSCs. We confirmed that CD133+ CSCs were presented in small percentages in several cancer cell lines cultured *in vitro*, mouse tumor xenografts and patients' tumor tissues. Furthermore, we confirmed that these CD133+ cancer cells were CSCs, which co-express TF and CD44. TF-targeting therapeutics, fVII-tPDT and ICON, could eradicate CSCs via induction of apoptosis and necrosis and via ADCC and CDC, respectively. These findings are consistent with our previous findings of the mechanisms of action for fVII-tPDT [15] and ICON [21] to kill cancer cells. When we initiated this project in 2008, CD133 was the major surface biomarker along with other commonly used markers such as CD44 for isolating CSCs [1]. Given that the CSC field evolved in the course of this study, CD24- and CD44-based protocols were used to validate the CD133-based protocols in the current work for isolation of breast CSCs. An earlier study showed that TF was expressed by CD133+ cancer cells that were isolated from a highly tumorigenic squamous cell carcinoma cell line A431 [28]. However, that earlier study did not include studies to ask whether those CD133+ A431 cells were cancer initiating stem cells, a characteristic that could be verified by tumorsphere assays *in vitro* and by a tumorigenesis assay *in vivo* [1]. Nonetheless, it provides independent evidence for TF expression in CD133+ cancer cells from a different cancer line.

An ideal surface target molecule for CSCs ought to be expressed selectively on CSCs, but not on normal cells, or even on some normal cells, these normal cells should be located outside of blood vessels and thus will be sequestered from direct contact with systemically administered therapeutic agents. We believe TF is such an ideal surface target for CSCs. Based on its selective expression and the leakiness of tumor neovasculature along with the findings in this study, we believe that TF is a common yet specific biomarker and therapeutic target not only for cancer cells and angiogenic vascular endothelial cells, but also for CSCs in tumor microenvironment. Although TF is expressed on extravascular cells and in the adventitial layer of the blood vessel wall under physiological conditions, it is sequestered from direct contact with circulating fVII and TF-targeting agents at these sites by the semipermeable normal blood vessels [29]. In contrast, newly formed tumor blood vessels are leaky because they lack tight junctions between endothelial cells, and their walls may even lack endothelial cells entirely [30, 31]. Thus, only the TF on CSCs, angiogenic VECs and cancer cells is accessible to TF-targeting therapeutic agents via leaky tumor blood vessels.

The finding that TF-targeted therapies are able to eradicate CSCs, in addition to their direct effects on cancer cells and tumor vessels, may help explain the marked *in vivo* efficacy of ICON and fVII-tPDT, which can eradicate established tumor xenografts (100-500 mm^3^) and prevent recurrence in preclinical mouse models of human prostate [14], melanoma [17], tongue [21] and lung [15] cancer. With respect to the levels required for complete tumor ablation, our previous murine studies showed that 50-100 ng/ml of plasma ICON levels in blood circulation was effective in eradication of distant metastasis after intratumoral injections of ICON-encoding adenoviral vectors [14]. In order to determine the optimal ICON concentration for humans, Phase I testing is needed. Delivery of ICON to the tumor target can be achieved by several means such as direct injection into tumors of ICON-encoded viral vectors [14, 17, 21] or i.v. administration of ICON protein [14]. These approaches have been detailed in previous publications [14, 17, 21, 32]. Regarding possible side effects, we have shown that ICON administration has minimal side effects in multiple animal models [13, 14, 17, 21]. Overall, the side effects of PDT using non-targeted photosensitizing agents are well described and manageable. Photosensitizing agents directed at TF would be expected to localize specifically to tumor cells and tumor vasculature. As has been shown in mouse models, TF-targeting with fVII-conjugated photosensitizers has the potential to significantly improve the selectivity and efficacy of PDT for the treatment of breast and lung cancer [15, 18–20]. On the other hand, this also makes it difficult to test the *in vivo* efficacy of TF-targeted therapies as a result of only eradicating CSCs alone, due to the observations that TF is expressed additionally on cancer cells and/or tumor vasculature *in vivo* in animal tumor models [13, 15]. Nevertheless, a newly developed cancer stem cell reporter vector system by the Wakefield group at NCI [33] may allow us to selectively label CSCs *in vitro* and *in vivo* in tumor xenografts and then to specifically examine *in vivo* evidence of the efficacy of TF-targeting therapies for eradication of CSCs in our ongoing studies using tumor cell line-derived and patient-derived xenograft mouse models.

To our knowledge, this study identifies for the first time that TF is a novel CSC therapeutic target and demonstrates that TF-targeted therapeutics (ICON and fVII-tPDT) can completely eradicate CSCs without drug resistance. In fact, our results indicate that CSCs are more sensitive than non-CSC cancer cells, to TF-targeting therapies. These findings may translate into other most commonly diagnosed solid cancer. This is because, similar to the cancer of breast, lung and ovary, TF is also expressed at high percentages in many other human solid cancers [15], for instance, 95% in primary melanoma and 100% in metastatic melanoma, 53%-90% in pancreatic cancer, 57%-100% in colorectal cancer, 63%-100% in hepatocellular carcinoma, 60%-78% in primary and metastatic prostate cancer and 47%-75% in glioma. Since CSCs represent a small population of malignant cells in a tumor tissue, targeting CSC alone therapy may not completely eradicate the established tumors. In this regard, TF-targeted therapeutics with ability to target three major tumor compartments, i.e., CSCs, cancer cells and tumor neovasculature, may achieve better efficacy and clinical outcome for cancer patients than those that only target CSCs, cancer cells or tumor angiogenesis/neovasculature alone.

## EXPERIMENTAL PROCEDURES

### Cell lines

Human lung cancer cell lines (H460 and A549) were cultured in RPMI-1640 (Invitrogen), human ovarian cancer lines (OVCAR5 and HEY) were cultured in F12 (Invitrogen), and the human triple negative breast cancer line (MDA-MB-231) was cultured in DMEM (Invitrogen), all supplemented with 10% heat-inactivated fetal bovine serum (FBS) (Sigma) and 1% penicillin/streptomycin (Invitrogen) at 37°C with 5% CO_2_. Patient-derived breast cancer cells and ADCC bioassay effector cells (a T cell-derived leukemia cell line Jurkat transfected with Fc receptor CD16 and NFAT-Luciferase under the control of CD16 binding followed by NFAT activation) (Promega) were cultured in RPMI-1640 with L-glutamine and HEPES (Gibco) supplemented with 10% heat-inactivated FBS, 1% penicillin/streptomycin, 0.1 mM MEM non-essential amino acids, 1 mM sodium pyruvate at 37°C with 5% CO_2_. The medium for ADCC effector cells was additionally supplemented with 250 μg/ml Antibiotic G-418 Sulfate Solution (Invitrogen) and 100μg/ml hygromycin (Invitrogen).

### Isolation of CD133+ CSCs from human cancer lines, tumor xenografts from mice and breast tumor tissues from patients

Using published procedures [34–36] [37–45] [28, 46–50] with modifications, CD133+ CSCs in this study were separately isolated using anti-CD133/1 (AC133) PE conjugate MACS microbead kit (Miltenyi Biotech) and EasySep PE positive selection kit (StemCell Technologies). For the MACS procedure, cancer cells were dissociated in non-enzymatic solution (Sigma) and were labeled with CD133/1 microbeads in the CD133 cell isolation kit (Miltenyi Biotech) by gently agitating for 30 min at 4°C. The single cell suspension was separated using a magnetic column (Miltenyi Biotech). The uncoated cells (CD133- cells) were removed by several washes with PBS/BSA (1× PBS pH7.4 free of Ca^2+^ and Mg^2+^, 1mM EDTA and 2% heat-inactivated FBS). The retained cells were designated CD133+ cancer cells.

We established the EasySep procedure for isolating CD133+ CSCs by modifying StemCell PE selection kit (StemCell Technologies) with PE-anti-CD133 antibody. After non-enzymatic dissociation, cancer cell suspension was incubated with PE-conjugated anti-human CD133/1 antibody (Miltenyi Biotech) at a concentration of 0.3-3μg/ml, were then incubated with EasySep PE Selection Cocktail and magnetic nanoparticles. Finally the cell suspensions were separated by magnet means. The uncoated cells (CD133- cells) were removed by four washes with PBS/FBS. The retained cells were designated CD133+ cancer cells.

When isolating CSC from tumor xenografts and tumor tissues from breast cancer patients, the tumor tissues were minced into small pieces (1-2 mm), washed twice in HBSS and then dissociated in HBSS with 1 mg/ml Collagenase IV (Sigma) for 30 min at 37°C with rotation. The dissociated cell suspension was filtered through a 70-mesh filter (Sigma). The cell suspension was washed in PBS and then processed for isolation of CD133+ cancer cells using the EasySep PE selection procedure or the MACS procedure, as described above. Note that isolation of cancer stem cells from surgically dissected tumor tissues from cancer patients has been approved by OSU IRB committee with patients' consent.

The protocol for isolating CD24-CD44+ CSCs was the same as suggested by the manufacturer using the CD24 CD44 breast cancer stem cell isolation kit (R&D) with a modification of using the EasSep magnet. The resulting three cell populations were termed CD24-CD44+ (CSCs), CD24-CD44- (non-CSCs) and CD24+ (non-CSCs) cells.

### Immunostaining and immunoblot analysis

After isolation, the CD133+ and CD133- cancer cells from the same parental cancer line were evaluated for expression of TF, CD133 and CD44 by immunofluorescent staining. Briefly, equal numbers (1 × 10^4^) of CD133+ and CD133-H460 cells were grown on the sterile 8-chamber slides for 48 hr, fixed with 4% paraformaldehyde for 15 minutes at room temperature. All slides were incubated with primary anti-human TF antibody (Clone HTF1, kind gift of Professor William Konigsberg), followed by incubation with FITC-conjugated anti-mouse IgG secondary antibody (Vector Laboratories). Then slides were incubated using PE-conjugated anti-human CD133/2 antibody (Miltenyi Biotech) or PE-conjugated anti-human CD44 antibody (BD Biosciences). Finally, the nuclei were counterstained with DAPI (Dilactate, Molecular Probes). Representative images were photographed using a LSM510 NLO confocal microscope (Carl Zeiss).

For immunoblotting, the isolated CD133+ and CD133- cancer cells were lysed in RIPA lysis buffer (Santa Cruz Biotech) and were electrophoresed in SDS-PAGE. The protein samples were transferred to a nitrocellulose (NC) membrane using Turbo Transfer unit (Bio-Rad). After blocking in 5% non-fat milk blocker (Bio-Rad), the NC membrane was incubated with 0.5 μg/ml monoclonal antibody against human TF (HTF1) followed by HRP conjugated a secondary antibody against mouse IgG (1:10,000, 1mg/ml stock, Vector Laboratories) and then with ECL reagents (Pierce) and exposure to film. After the first probing for TF, the NC membrane was incubated in Western stripping buffer (Pierce) to sequentially re-probe with anti-CD133/1 antibody (Miltenyi Biotech) (0.5 μg/ml stock, 2^nd^ probing) for CD133 followed by anti-mouse IgGFc HRP conjugate (Vector Laboratories) and then with anti-GAPDH HRP (1:5000) (Novus Biologicals) for assessing sample loading.

### Tumorsphere assay *in vitro* in matrigel microplates

The tumorsphere assay was done as described [39]. Briefly, 96-well plates were pre-coated with 50 μl Matrigel (BD Biosciences) per well, then CD133+ and CD133- cancer cells were suspended in growth medium supplemented with 10% FBS (Gibco) or Mammocult^TM^ Basal medium (Stemcell Technologies) and seeded upon Matrigel at the density of 100, 50, 25 or 12.5 cells per well. All experiments were conducted in triplicate wells. The numbers of colony were counted at week 1, 2 and 3. The data were presented as mean ± SEM and representative views were photographed.

### *In vivo* tumorigenicity of CD133+ and CD133- cancer cells in mice

The *in vivo* tumorigenicity of isolated CD133+ and CD133- cancer cells was assessed and compared as formation of tumors in severe combined immunodeficiency (SCID) mice. After isolation, the CD133+ or CD133- cancer cells were mixed with an equal volume of Matrigel (BD Biosciences) and 50 μl of cancer cells/Matrigel mixture containing 100, 200, 500 and 1,000 CD133+ or CD133- lung cancer cells were subcutaneously injected into right and left flanks of 3 weeks male SCID/Beige mice (Taconic Farms). The tumor volume was calculated by measuring tumor width and length in millimeters (mm) with calipers twice a week from day 0 to day 38 and using tumor volume (mm^3^)=(width)^2^ × length/2, as described [13, 14, 17, 51]. Photographs of mice and tumors were taken during and at the time of sacrifice of the mice. At the end of experiments, CD133+ and CD133- cancer cell-derived tumors were dissected and weighed separately.

### Product of fVII-SnCe6 conjugate

A recombinant protein consisting of murine factor VII (mfVII) with a K341A mutation followed by two short amino acid tags, S peptide with D14N mutation (S tag) and 6x Histidine tag (His tag) in the C-terminus was produced using recombinant DNA technique, as described [15, 20]. After affinity purification using Ni-NTA (Qiagen), fVII protein was characterized and conjugated to Sn(IV) Chlorin e6 dye (Frontier Scientific) by using a cross linker EDC (Pierce). After size exclusion separation of fVII conjugated dye from free dye, fVII-SnCe6 conjugate was verified for binding activity to cancer cells by ELISA and then was used for fVII-tPDT below.

### fVII-tPDT for CD133+ and CD133- cancer cells *in vitro* and crystal violet membrane staining and clonogenic assays

The procedure of fVII-tPDT *in vitro* using fVII-SnCe6 conjugate was described in detail [15, 20]. Briefly, 1 × 10^4^ cells of CD133+ CSCs, CD133- and parental cancer cells were seeded in 96-well plate overnight, washed once with 1X HBSS containing 1% BSA, incubated with fVII-SnCe6 in 1X HBSS containing 1% BSA and 10 mM CaCl_2_, or the buffer only as non-treatment control, for 90 minutes as optimized drug light interval [20]. The cells were irradiated with 635 nm fiber delivery diode laser with adjustable power (0-250 mW/cm^2^, B & W Tek, Inc) at different energy. The PDT effect was determined by the monolayer cell membrane staining with crystal violet and by a clonogenic assay, as described [15, 20, 52]. The therapeutic effect was presented as cell survival percentage (%) based on OD595 nm using the formula: cell survival percentage (%)=(PDT treated-maximal killing control)/(untreatment control-maximal killing control) × 100%. In the conventional clonogenic assay, the colony numbers were counted by staining the colonies with crystal violet and the cell survival %= PDT treated/untreated control × 100%.

### Assays for apoptosis and necrosis

After CD133+ CSCs and non-CSCs were treated by fVII-tPDT, markers for apoptosis and necrosis were assayed using ApoDETECT Annexin V-FITC kit (Invitrogen), in which Annexin V FITC staining for apoptosis and propidium iodide staining DNA for necrosis, as described [15].

### ICON-mediated ADCC assay

ICON-mediated ADCC to CSCs and non-CSCs was determined using an ADCC effector assay (Promega) [53] following the manufacturer's instruction with a critical modification to the assay medium by using DMEM medium supplemented with 0.5% super low IgG FBS (HyClone), instead of RPMI 1640. The reason is that DMEM medium contains relatively a higher calcium concentration (1.8 mM) than RMPI 1640 (0.42 mM) according to the information on calcium in cell culture from Sigma-Aldrich. Calcium is required for ICON and fVII binding to its receptor TF [13]. Briefly, CSCs and non-CSC cancer cells (4 × 10^3^ or 1 × 10^4^ cells per well) were seeded in duplicate into the 60 inner wells and grown overnight in 96-well white assay plate (Corning) at 37°C and 5% CO_2_. The next morning ICON protein at serial dilutions was added in duplicate wells to CSCs and non-CSC cancer cells, whereas human IgG (Sigma) was used as IgG isotype control. After 15 min incubation at 37°C, ADCC effector cells were added to achieve a ration of effector to target (E:T) at 25:1. At the end of 6-hr or 19-hr incubation, equal volume of Bio-Glo assay reagent (Promega) was added and raw bioluminescence (RLU) was read on SpectraMax i3 (Molecular Devices) every 5 min for 30 min and analyzed by Prism software.

### ICON-mediated CDC assay

ICON-CDC to CD133+ CSCs and CD133- non-CSCs was determined by a fluorescent dye Calcein AM release assay, as described [21]. Briefly, the CD133+ CSCs and non-CSC CD133- cells were seeded in 96-well U-bottom microplates overnight. The next morning the cells were labeled with 4 μM Calcein AM (Molecular Probes) for 40 min and then the cells were incubated with ICON diluted in 1× VBS buffer supplemented with 10mM CaCl_2_ and followed by incubation with 1:4 diluted rabbit complement MA (Cedarlane Laboratories) as a source of complements. Controls include ICON alone and complement alone, buffer alone (as spontaneous release control) and maximal release control (0.1% Triton X-100 lysis). After 4-hr incubation, the plate was briefly centrifuged and supernatant was transferred to a new 96-well black plate with clear, flat bottom. Fluorescence in supernatants was read on a fluorescence microplate reader (Molecular Devices). CDC cytotoxicity was calculated as described [21].

### Statistical analysis

All the data *in vitro* and *in vivo* are presented as mean **±** SEM and analyzed for statistical significance using SAS 9.4 software. Linear mixed effects models and t-tests were performed for comparing CD133+ and CD133- groups. Multiple comparisons were adjusted by Holm's method where appropriate. For analyses of statistical significance triplicate or quadruplicate wells in each group were used for tumorsphere assays *in vitro* in tissue culture plates and 3 mice per group were used *in vivo* in animal studies. The significance of tumorigenicity between CD133+ CSCs and non-CSC CD133- cancer cells was determined by comparing their tumor volumes side by side in the same mice (3 mice per group) using ANOVA. *: p<0.05; **: p<0.01; ***: p<0.001; ****: p<0.0001.

## SUPPLEMENTARY FIGURES AND TABLES



## Abbreviations

CSCS: Cancer stem cells
TF: Tissue factor
FVII: coagulation factor VII
ICON: fVII(K341A)-IgG1Fc immunoconjugate
PS: Photosensitizer
PDT: Photodynamic therapy
fVII-tPDT: factor VII-targeted photodynamic therapy
CD: Cluster of differentiation
ADCC: Antibody-dependent cell-mediated cytotoxicity
CDC: Complement-mediated cytotoxicity
TNBC: Triple-negative breast cancer
VEC: Vascular endothelial cells
AMD: Age-related macular degeneration.
