# Supplementary material for: Targeting tissue factor as a novel therapeutic oncotarget for eradication of cancer stem cells isolated from tumor cell lines, tumor xenografts and patients of breast, lung and ovarian cancer
Source: Oncotarget. 2016 Nov 26;8(1):1481–94. doi: 10.18632/oncotarget.13644 (PMC5352071; doi:10.18632/oncotarget.13644)
Supplement: Supplementary file 1 [file oncotarget-08-1481-s001.pdf]

Targeting tissue factor as a novel therapeutic oncotarget for eradication of cancer stem cells isolated from tumor cell lines, tumor xenografts and patients of breast, lung and ovarian cancer

SUPPLEMENTARY FIGURES AND TABLES

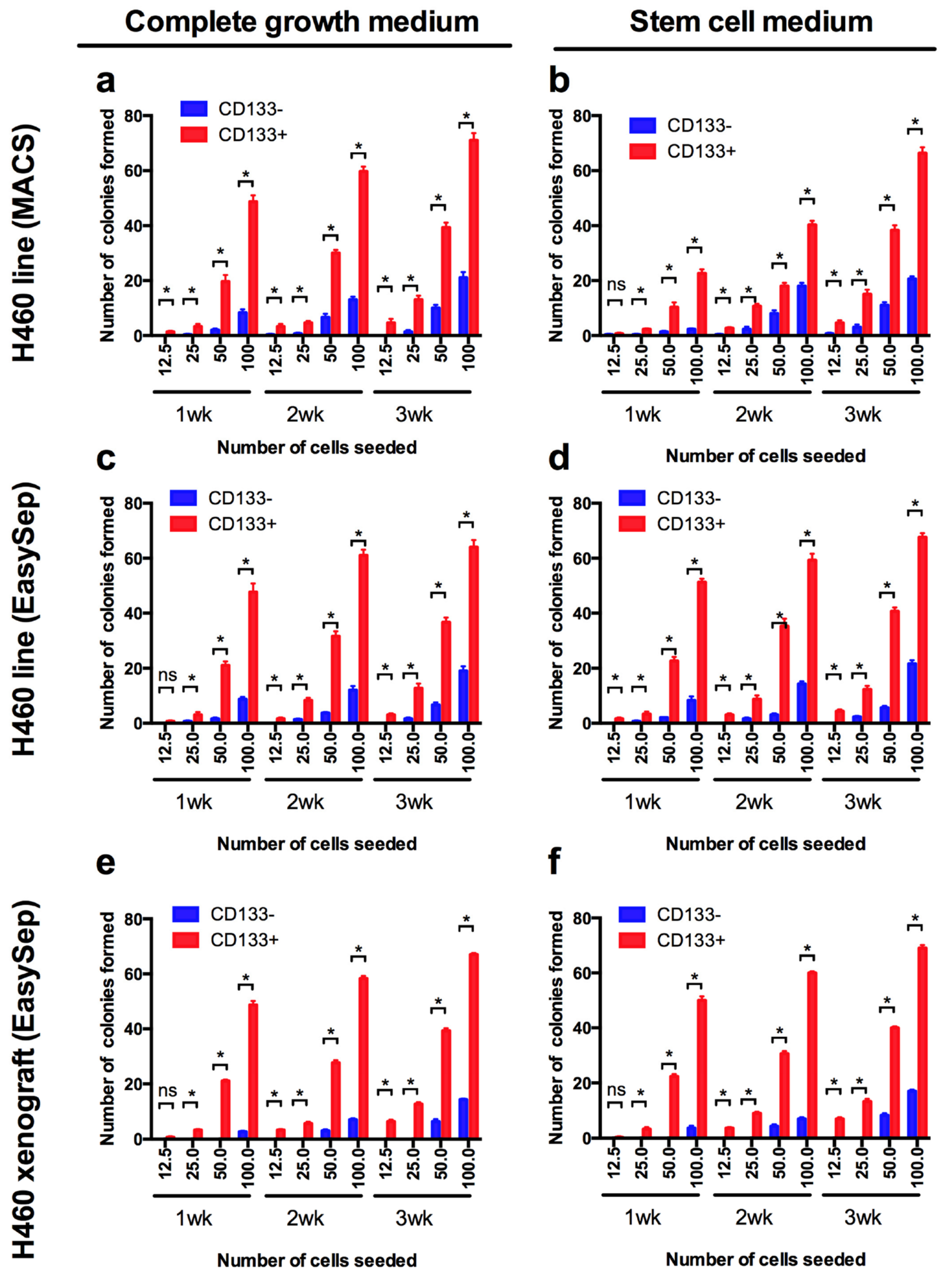

Supplementary Figure S1: Tumorsphere assays for CD133+ and CD133- cancer cells at week one, two and three. After isolation by using the MACS protocol from the H460 line **a & b.** or by using the EasySep protocol from the H460 line **c & d.** or from H460 tumor xenograft **e & f.,** CD133+ and CD133- cancer cells were seeded with 100, 50, 25 and 12.5 cells through serial dilutions onto Matrigel basal layer in triplicates in 96 well plates and grown in serum containing complete growth medium (left column) or in Mammocult stem cell serum free medium (right column). All *p* values were analyzed for the tumorsphere numbers between CD133+ and CD133- cells at the same seeding numbers. *: $p<0.05$. ns: *p* value not significant.

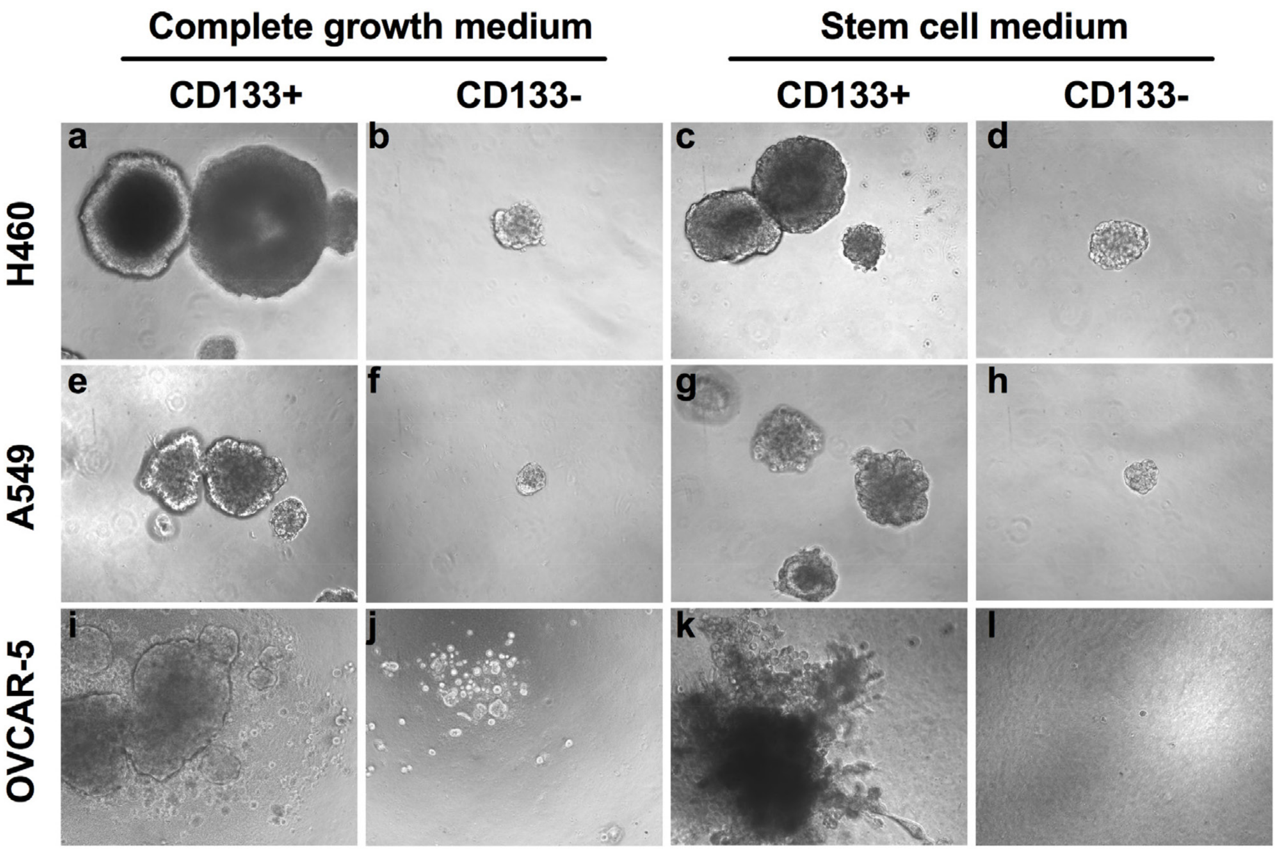

Supplementary Figure S2: CD133+ cancer cells are CSCs as they are capable of forming more and larger tumorspheres than CD133- cancer cells in a tumorsphere assay, a model of *in vitro* tumorigenicity as a characteristic of cancer stem cells. After isolation, CD133+ and CD133- cancer cells were seeded onto Matrigel basal layer in triplicates and grown in serum containing complete growth medium (**a & b.** for H460, **e & f.** for A549, **i & j.** for OVCAR-5) or in Mammocult stem cell serum free medium (**c & d.** for H460, **g & h.** for A549, **k & l.** for OVCAR-5) in 96 well plates. The tumorspheres were photographed at week 3 (a-j) or week 1 (k & l) and the sphere numbers were summarized and analyzed in Supplementary Table S2 and Supplementary Figure S3. Original magnification: 200 ×.

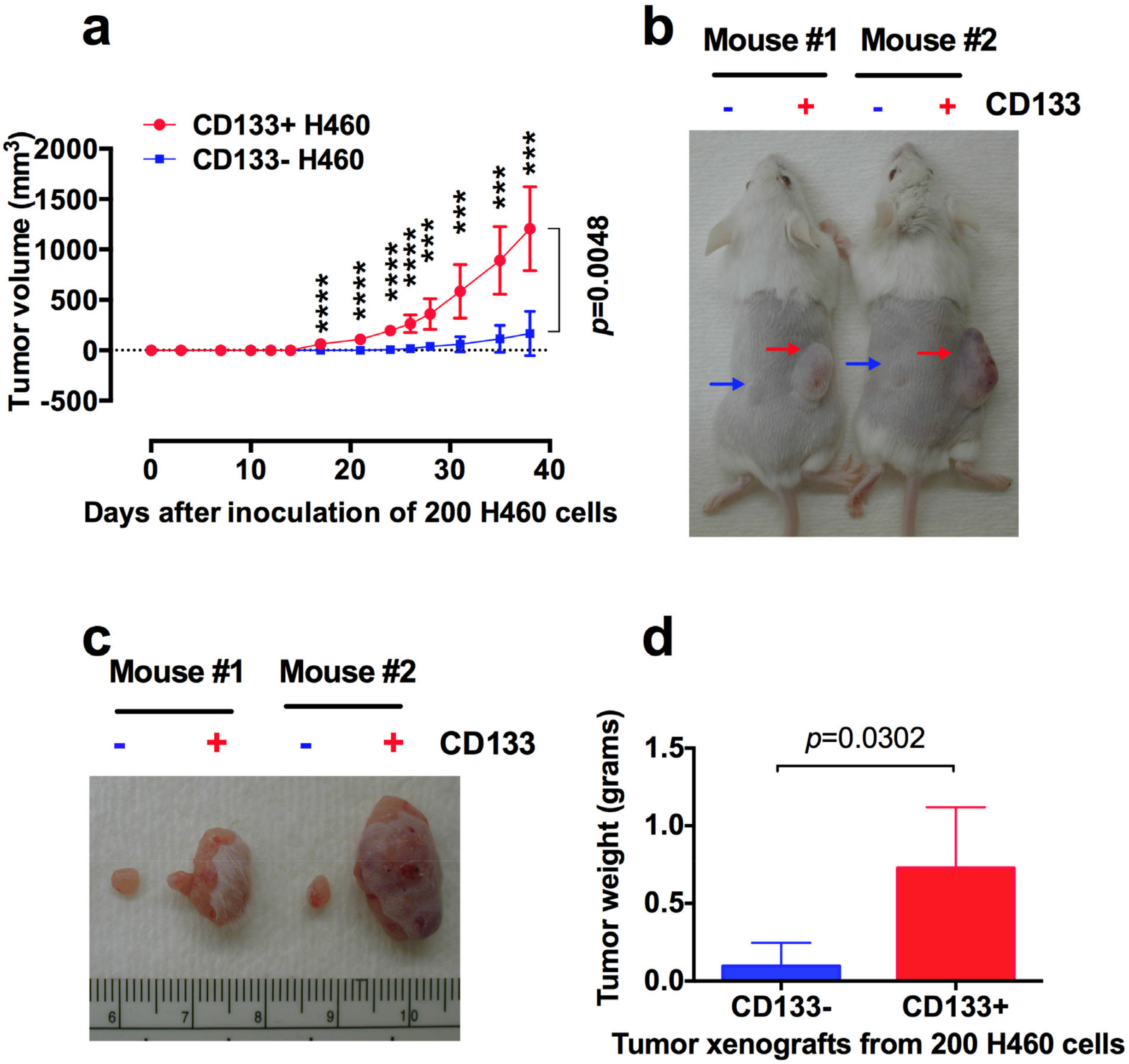

Supplementary Figure S3: CD133+ cancer cells are CSCs as they are capable of forming larger tumors and earlier *in vivo* in mice than CD133- cancer cells in an *in vivo* tumorigenicity assay. a-d. *In vivo* xenograft formation from 200 CD133+ and CD133- H460 cells at right and left flanks, respectively, in SCID Beige mice. a. Tumor growth curves (Mean ± SEM). ****: *p<0.0001 or* ***: *p<0.005* from days 17-38 and *p=0.0048* on day 38 by linear mixed model with t-test. b. Morphology of the mice bearing CD133 positive (red arrows) and CD133 negative (blue arrows)-derived tumors; c. Tumor dissected from mice ("-" for CD133 negative and "+" for CD133 positive cells); d. Tumor weights (*p*=0.0302, one-tailed unpaired t test).

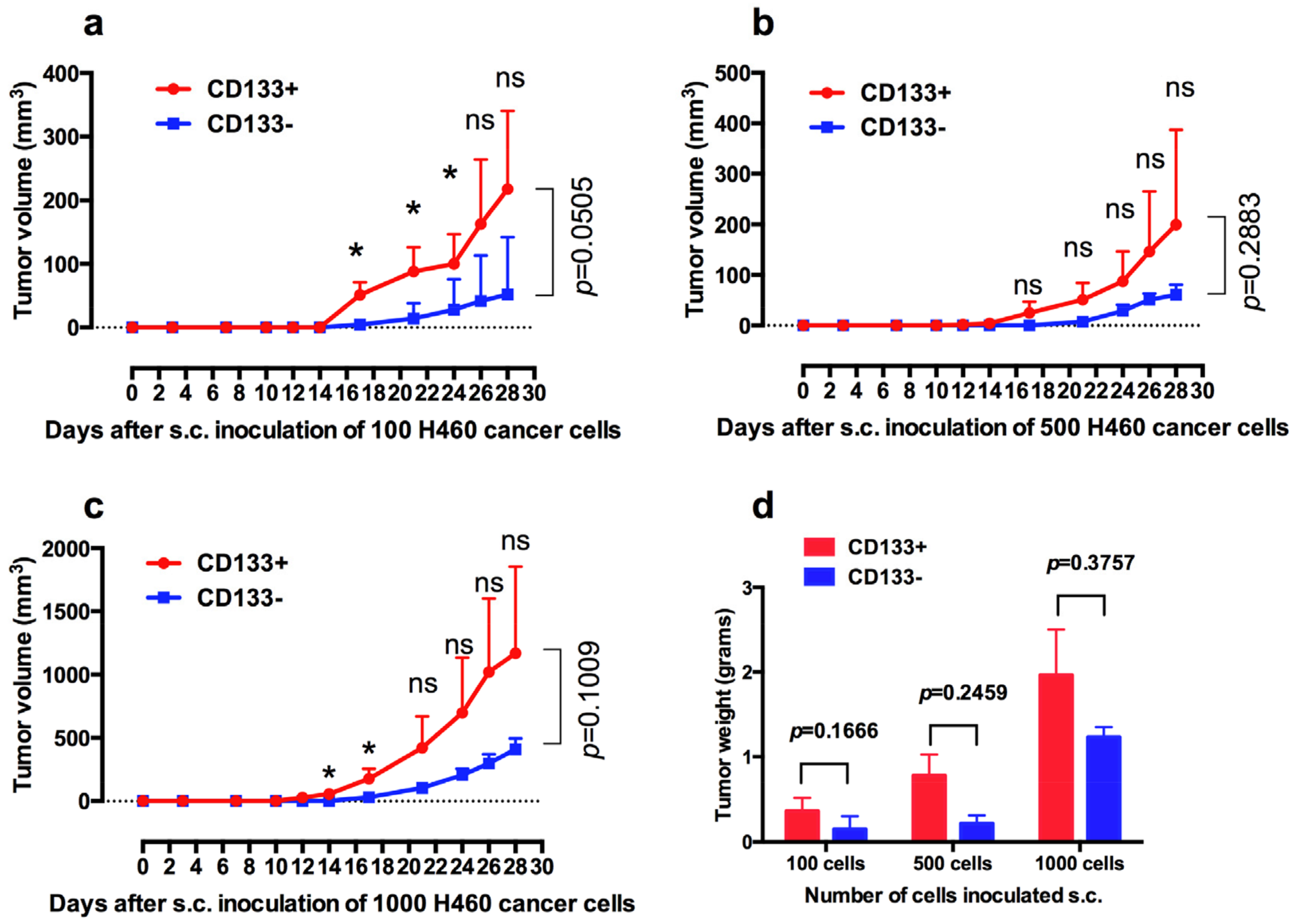

Supplementary Figure S4: CD133+ cancer cells are capable of forming larger tumor and earlier than CD133- cancer cells *in vivo* in SCID Beige mice. After isolation, CD133+ and CD133- H460 cancer cells were injected subcutaneously with 100, 200, 500 and 1000 cells mixed with Matrigel at right and left flanks, respectively, in SCID Beige mice. The results with 200 cells were shown above in Supplementary Figure S3. The results of tumor xenograft growth with 100 cells **a.**, 500 cells **b.** and 1000 cells **c.** are shown here. Tumor weights are shown in panel **d.** (*p* values for CD133+ derived tumors vs. CD133- derived tumors). *: $p<0.05$. ns: not significant.

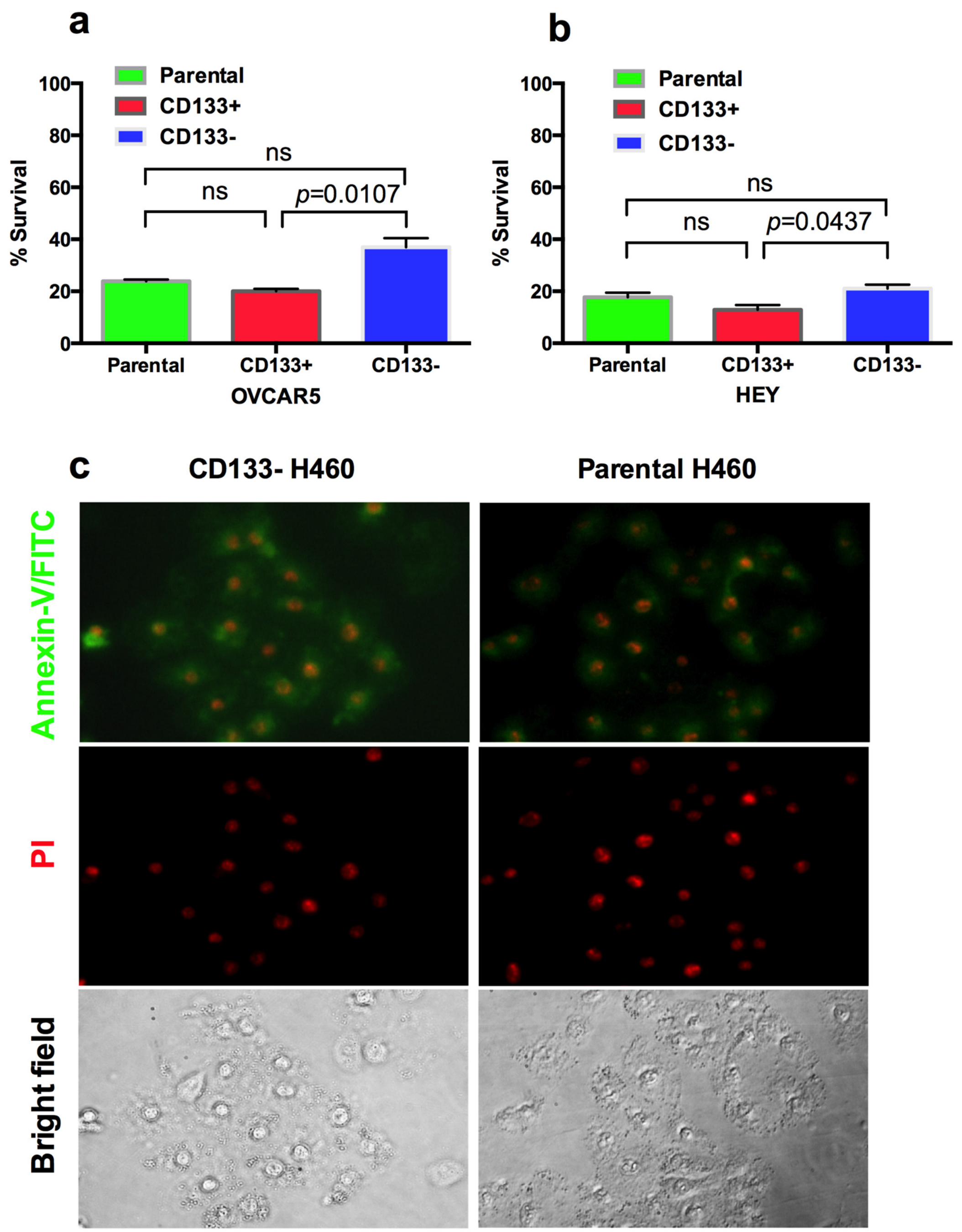

Supplementary Figure S5: The Effect and mechanism of actions of fVII-tPDT in eradicating CD133+, CD133- and parental ovarian and lung cancer cells *in vitro*. CD133+, CD133- and parental ovarian cancer cells OVCAR-5 **a.**, HEY **b.** and H460 cells **c.** were seeded in 96 well plates with 2×10^4 cells per well. Next day, the cells were treated by fVII-tPDT at 2 µM SnCe6 for 36 J/cm^2 635nm laser light and the therapeutic effect was determined by membrane staining assay (see the details in Experimental Procedures). *: *p* values were significant between CD133+ CSCs and CD133- non-CSC cancer cells. ns: not significant. (c) After fVII-tPDT treatment, the CD133- and parental H460 cells were stained with 1:20 diluted Annexin V-FITC for early detection of apoptotic cells and then stained with 1µg/ml Propidium Iodide (PI) for necrotic cells using ApoDETECT ANNEXIN V-FITC KIT (Invitrogen). After staining, the cells were observed and photographed under a fluorescent microscope using green (FITC), red (PI), and phase channels. Original magnification: 200 ×

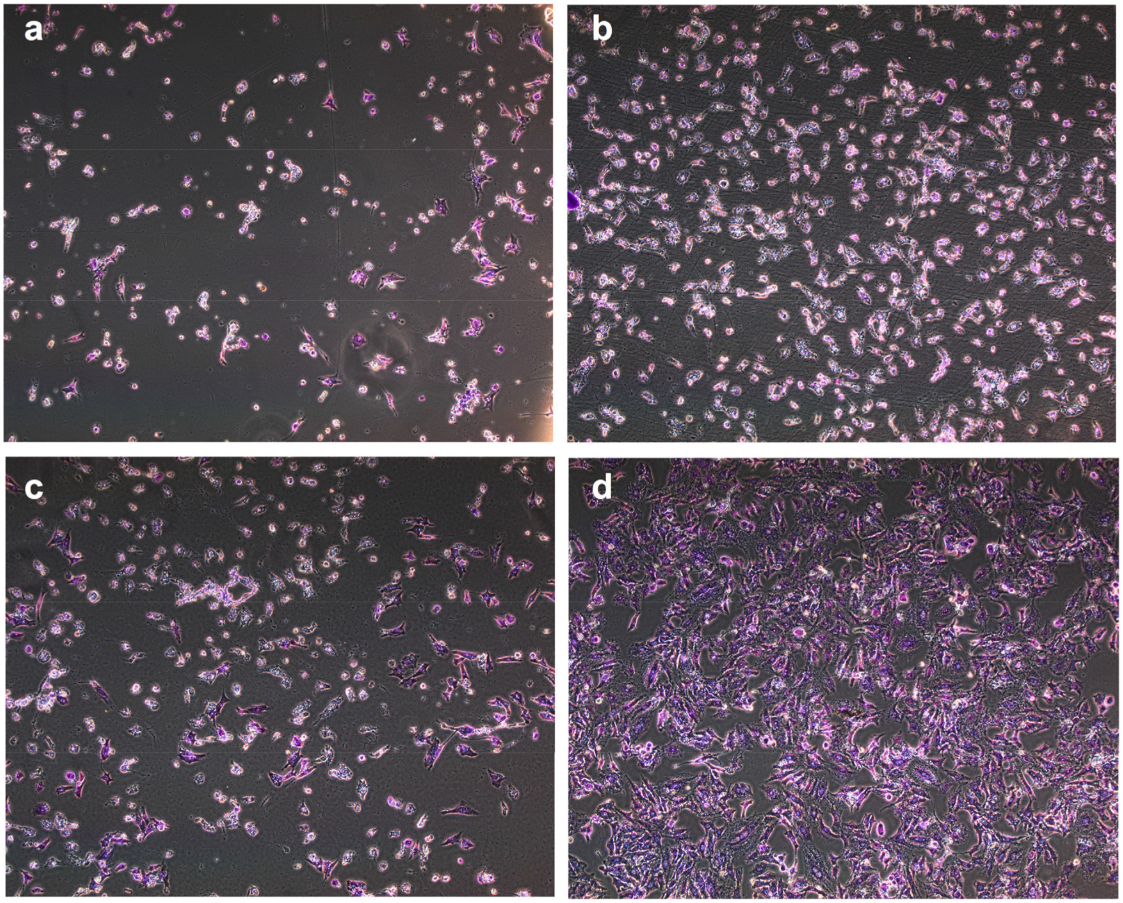

Supplementary Figure S6: Crystal violet-stained CD133+ CSC, CD133- non-CSC, parental and untreated human lung cancer A549 cells after fVII-tPDT treatment. After fVII-tPDT, CD133+ CSC **a.**, CD133- non-CSC **b.**, parental **c.** and untreated human lung cancer A549 cells **d.** were fixed and stained with crystal violet, as described in the Methods. The cells were photographed before being de-stained for OD595 nm reading in the monolayer-membrane staining assay. Original magnification: 100 ×.

Supplementary Table S1: Percent of CD133+ cancer cells isolated from cultured cancer lines *in vivo* and tumor xenografts *in vivo* in mice

Cancer type	Cancer lines, xenografts and patients	*CD133+ cancer cells % by EasySep	*CD133+ cancer cells % by MACS	***p* values (EasySep vs. MACS)
**Lung cancer**	H460 line [a]	0.864 ± 0.196 (n=5)	0.690 ± 0.190 (n=2)	0.6343
	H460 xenograft [b]	0.535 ± 0.015 (n=2)		0.5655 (a vs. b)
**Ovarian cancer**	OVCAR-5 line	0.703 ± 0.486 (n=3)	0.607 ± 0.272 (n=3)	0.8705
	HEY line	0.025 ± 0.005 (n=2)	0.100 (n=1)	
**Breast cancer**	MDA-MB-231 line [c]	2.100 ± 1.479 (n=4)		ns (c vs. d)
	MDA-MB-231xenograft [d]	3.267 ± 1.598 (n=3)		ns (d vs. e)
	Breast cancer patients [e]	3.820 ± 2.429 (n=5)		ns (c vs. e)

* CD133+ % is presented as Mean ± SEM; ** ANOVA and unpaired t-test; ns, not significant.

Supplementary Table S2: Colony formation by CD133+ and CD133- cancer cells isolated from human lung, ovarian and breast cancer cell lines and tumor xenografts from mice

Cancer type	Number of cells seeded	Tumorspheres in growth medium			Tumorspheres in stem cell medium			CD133 PE-based methods
		Number of tumorspheres formed (mean ± SEM)		*p* values* (CD133+ vs. CD133-)	Number of tumorspheres formed (mean ± SEM)		*p* values* (CD133+ vs. CD133-)	
		CD133+	CD133-		CD133+	CD133-		
**Lung cancer line (H460) (11-22-09) MACS**	12.5	3.3 ± 0.9	0.3 ± 0.3	0.0058	2.7 ± 0.3	0.3 ± 0.3	0.0001	MACS
	25	4.7 ± 0.7	0.7 ± 0.3	<0.0001	10.7 ± 0.9	2.3 ± 0.9	<0.0001	
	50	30.0 ± 1.2	6.7 ± 1.2	<0.0001	18.0 ± 1.2	8.0 ± 1.2	<0.0001	
	100	59.7 ± 1.8	13.0 ± 1.2	<0.0001	40.3 ± 1.5	18.0 ± 1.2	<0.0001	
**Lung cancer line (H460) (2-4-10) EasySep**	12.5	1.7 ± 0.3	0.0 ± 0.0	0.0001	3.0 ± 0.6	0.0 ± 0.0	<0.0001	EasySep
	25	8.3 ± 0.9	1.3 ± 0.3	<0.0001	8.7 ± 1.5	1.7 ± 0.3	0.0002	
	50	31.7 ± 1.8	3.7 ± 0.3	<0.0001	35.3 ± 2.7	3.0 ± 0.6	<0.0001	
	100	61.0 ± 2.1	12.0 ± 1.5	<0.0001	59.3 ± 2.3	14.3 ± 0.9	<0.0001	
**Lung cancer xenograft (H460 8-11-10) EasySep**	12.5	3.3 ± 0.3	0.0 ± 0.0	<0.0001	3.7 ± 0.3	0.0 ± 0.0	<0.0001	EasySep
	25	5.7 ± 0.7	0.0 ± 0.0	<0.0001	9.0 ± 0.6	0.0 ± 0.0	<0.0001	
	50	27.7 ± 0.9	3.0 ± 0.6	<0.0001	30.7 ± 0.9	4.3 ± 0.7	<0.0001	
	100	58.3 ± 0.9	7.0 ± 0.6	<0.0001	60.0 ± 0.6	7.0 ± 0.6	<0.0001	
**Lung cancer xenograft (A549) EasySep**	12.5	0.7 ± 0.3	0.0 ± 0.0	0.0628	0.7 ± 0.3	0.0 ± 0.0	0.0628	EasySep
	25	3.7 ± 0.3	0.0 ± 0.0	<0.0001	4.0 ± 0.6	0.0 ± 0.0	<0.0001	
	50	20.3 ± 0.3	0.0 ± 0.0	<0.0001	23.3 ± 0.9	0.0 ± 0.0	<0.0001	
	100	45.7 ± 1.2	2.3 ± 0.3	<0.0001	49.3 ± 0.9	3.3 ± 0.3	<0.0001	
**Ovarian cancer line (OVCAR-5) (11-6-09)**	12.5	2.3 ± 0.3	0.0 ± 0.0	<0.0001				EasySep
	25	10.3 ± 0.9	1.0 ± 0.6	<0.0001				
	50	35.7 ± 2.3	3.0 ± 1.2	<0.0001				
	100	68.0 ± 3.1	13.3 ± 1.5	<0.0001				
**TNBC line** (MDA-MB-231)**	10	3.3 ± 0.9	0.0 ± 0.0	0.0030				EasySep
	20	15.0 ± 3.9	0.0 ± 0.0	0.0011				
	50	28.5 ± 3.2	0.0 ± 0.0	<0.0001				

Multiple t tests (Prism version 6.0h); ** Tumorspheres were counted at one week. All other data in Table 2 were analyzed from two weeks results. N/A: Not available. TNBC: Triple-negative breast cancer.

Supplementary Table S3: Effect of fVII-tPDT in killing CSC *in vitro* determined by clonogenic assays

Cancer type	SnCe6 (μM) in fVII-tPDT (36J/cm^2)	H460			A549		
		% Cell survival (mean ± SEM)		*p* values* (CD133+ vs. CD133-)	% Cell survival (mean ± SEM)		*p* values* (CD133+ vs. CD133-)
		CD133+	CD133-		CD133+	CD133-	
**Lung cancer**	0.0	100.0 ± 0.5	100.0 ± 5.5	ns	100.0 ± 1.8	100.0 ± 0.0	ns
	0.1	74.3 ± 4.0	89.8 ± 2.7	ns	76.7 ± 1.1	96.1 ± 1.3	****
	0.5	11.4 ± 1.1	29.7 ± 0.6	**	0.8 ± 0.07	84.2 ± 5.3	****
	1.0	0.004 ± 0.001	0.3 ± 0.001	***	0.0 ± 0.0	0.3 ± 0.09	ns
	2.0	0.0 ± 0.0	0.0 ± 0.0	ns	0.0 ± 0.0	0.0 ± 0.0	ns

Notes: ns: not significant; **: <0.01; ***: <0.001; ****: <0.0001.

Supplementary Table S4: Comparison of clonogenic assay and crystal violet membrane staining for determining the effect of fVII-tPDT in killing CD133+, CD133- and parental H460 lung cancer cells

Assays	Clonogenic assay			Crystal violet membrane staining		
	**CD133+**	**CD133-**	**parental**	**CD133+**	**CD133-**	**parental**
**% Survival ***	0.0 ± 0.0	0.0 ± 0.0	0.09 ± 0.06	30.8 ± 1.8	39.4 ± 1.3	45.1 ± 0.2
p* values*	CD133+ vs. CD133-: 1.0000			CD133+ vs. CD133-: 0.0171		
	CD133+ vs. parental: 0.1462			CD133+ vs. parental: 0.0041		
	CD133- vs. parental: 0.1462			CD133- vs. parental: 0.0499		

Notes: * % survival presented as mean ± SEM. ** *p* values were analyzed using 2way ANOVA (Prism version 6.0h). (fVII-PDT: 2 μM SnCe6 in fVII-SnCe6, 36 J/cm^2, 635nm laser).
